# Spatio-seasonal modeling of the incidence rate of malaria in Mozambique

**DOI:** 10.1186/1475-2875-7-228

**Published:** 2008-10-31

**Authors:** Rosa Abellana, Carlos Ascaso, John Aponte, Francisco Saute, Delino Nhalungo, Ariel Nhacolo, Pedro Alonso

**Affiliations:** 1 Bioestadistica. Departament de Salut Publica, Universitat de Barcelona, Casanova 143, 08036, Barcelona, Spain; 2 Centro de Salud Internacional, Institut d'Investigacions Biomediques Agust Pi i Sunyer. IDIBAPS, Barcelona, Spain; 3 Centro de Investigaao em Saude da Manhiça (CISM), Ministério de Saúde, Manhiça, Maputo, Moçambique

## Abstract

**Background:**

The objective was to study the seasonal effect on the spatial distribution of the incidence of malaria in children under 10 years old living in the Manhiça district, Mozambique.

**Methods:**

The data of the clinical malaria incidence were obtained from a study of two cohorts of children followed from December 1996 to July 1999. The cases were obtained by the active detection method. Hierarchical Bayesian models were used to model the incidence of malaria, including spatial correlation nested to climatic season. The models were compared with the deviance information criterion. The age and gender of the children were also taken into account.

**Results:**

The incidence of malaria is associated with age, period and climate season. The incidence presents a clear spatial pattern, with a higher incidence in the neighbourhoods situated in the north and northeast of the Manhiça area. The transmission of malaria is highest during the wet season but the spatial pattern of malaria does not differ from that during the dry season.

**Conclusion:**

The incidence of malaria in Manhiça presents a spatial pattern which is independent of the seasonal climatic conditions. The climate modifies the incidence of malaria in the entire region but does not change the spatial pattern of the incidence of this disease. These findings may be useful for the planning of malaria control activities. These activities can be performed taking account that the neighbourhoods with more incidence of malaria do not change over the annual climate seasons.

## Background

Malaria has been and still is the cause of much human morbidity and mortality. Although the disease has been eradicated in most temperate zones, it continues to be endemic in most of the countries of sub-Saharan Africa and particularly, in the area of Mozambique where this study was performed. The study of malaria incidence conducted by Saute *et al *[1] in the area of Mozambique supports the hypothesis of the existence of zones with higher levels of malaria risk. The Mapping Malaria Risk in Africa (MARA) collaboration [2] located the Manhiça area as a zone with seasonal and endemic malaria risk, with transmission being maximum between November and April.

There are natural and social factors affecting the dynamics of the transmission of infection. Temperature and rainfall are considered major natural risk factors that affect the cycle's life and the breeding of the mosquitoes that transmit the malaria (*Anopheles*). In this area of study the main species is *Anopheles funestus *(about 72.3% of the anopheline population [3]). The eggs of the mosquito can survive under certain climatologic conditions, mainly humidity and warm temperature [4]. A reasonable amount of rainfall can create additional breeding sites for mosquitoes, increasing their population, and extending the radius of infection. Temperature may affect the life expectancy of the mosquito and the behaviour of the human host. Thus, the variation in seasonal climatic conditions can change the spatial distribution of the incidence of malaria. To study the spatial distribution of the disease, Besag [5] proposed the conditionally autoregressive model that accounts for spatial similarities among nearby regions. Later, Besag, York and Mollié [6] proposed a model for separating the spatial effects from the overall heterogeneity in the rates. To incorporate the effect of the climatic season over the spatial variability of the rates around the neighbourhoods, the spatio-temporal model described by Waller *et al *[7] is used, changing the temporal concept for the seasonal concept. This allowed us to define nested random effects, wherein the spatial effects are nested within the climatic season. In this way, the impact of the climatic seasons on the spatial patterns of the incidence of malaria could be evaluated. The conditionally autoregressive model is used with observations at regions of space (i.e. the analysis of aggregate count data per region in the context of disease mapping [8]) and the spatial correlation is usually defined among regions which are geographically closed. However, geostatistical methods most often work with observations at points of space with the objective of making a statistical interpolation to a field or continuous surface assumed to extend across the whole study area. In these methods the spatial correlation is based on the distance or combination the distance and the location [9].

The main aim of this study was to evaluate whether the spatial patterns of the incidence of malaria in children under the age of 10 years living in an endemic area of the Manhiça district (in southern Mozambique) vary between the wet and dry seasons. The variables of age and sex were also considered in the analysis.

## Methods

### Study area and population

The study was performed in the Manhiça district (Province of Maputo) in Southern Mozambique where the Centro de Investigaçao Saúde de Manhiça (CISM) is located.

**Figure 1:**
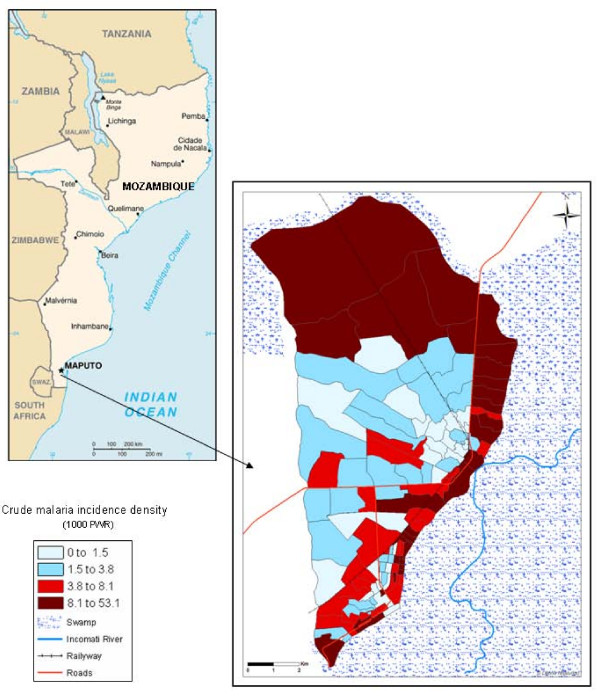
**Geographic distribution of crude malaria incidence rate in the Manhiça study area (Province of Maputo, Mozambique).** The study area is divided into 115 administrative neighbourhoods (map of the right side). This map shows the crude malaria incidence for each neighbourhood. The incidence density is expressed as 1000 children week at risk (PWR).

**Figure 2:**
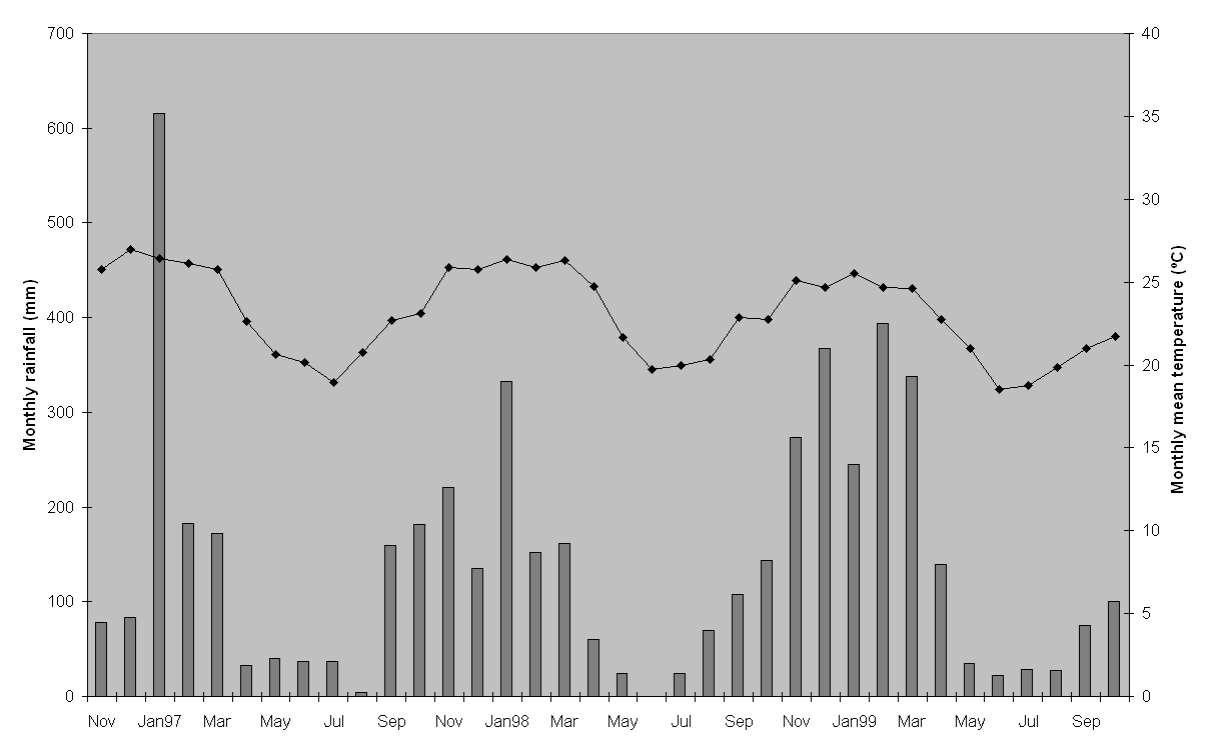
**Meteorological data.** Monthly rainfall (bars) and monthly mean temperatures.

The village of Manhiça, 25° 24'S and 32° 48'E, is situated in an area of flat bush costal savannah along the coast of Mozambique, 80 km north of the capital Maputo. The village has an approximate extension of 120 km^2^. It is located in a well-drained sandy elevate plane, surrounded by the Incomati River meanders (Figure 1). The lower river floodplain supports anopheline larval breeding all year. Houses are typically constructed with reeds and have thatched or corrugated roofs. Most of the population inhabits the elevated area, and the lower plane is used for crops. The climate is subtropical with two principal seasons, one warm and rainy lasting from November to April, defined as transmission malaria season by the MARA project [2], and one cold and dry over the remaining months [1]. The annual rainfall was about 1,500 mm during the study period. The average temperature was about 23°C, whereas the mean temperature for June and July is around 19°C and for January between 26 and 27°C (Figure 2). The temperature and the rainfall were recorded by the climatology station located near CISM.

The area was subdivided using its political-administrative division and 115 neighbourhoods were obtained. This area is under demography surveillance since 1996 [10]. The population of this area is commonly peri-rural or rural, and most of the families make a living working in cooperatives of sugar, bananas and rice. This area has one hospital, the Manhiça Health Centre with 80 beds. Finally, this area has a main road and a railway which crosses the area from south to north.

The source of maps of the area was the Direcção Nacional de Geografia e Cadastro of Mozambique (Numbers of reference: SG-36/I-I-NE page 1169 2532, SG-36/J-I-NO page 1170 2533A1, SE-26/V-III-SO page 1178 2532B3, SG-36/I-IV-SO page 1179 2532B4). The maps were scanned and georeferenced the limit of the neighbourhoods by CISM.

### Study subjects and covariate data

Data on the incidence of clinical malaria episodes were obtained from a study performed by the CISM in this area [1]. Two cohorts of children were studied. The first cohort was a random sample stratified by age of children less than 10 years old followed from December 1996 to July 1999. The second cohort, which complemented the first, was composed of the children born in each year of the study. The children under study had to reside in the study area for at least three months, and in the case of newborns, the parents had to have lived there for three or more months. Both cohorts were identically followed and each child was visited at home once a week by a trained fieldworker.

Each child visit was counted as one-person week at risk (PWAR). Clinical malaria was defined as fever (with an auxiliary temperature greater than or equal to 37.5C°) with a positive blood slide (with one or more asexual *Plasmodium falciparum *parasites). The children diagnosed with malaria were treated and excluded from the following period of 28 days. This study falls within the National Ethical Clearance granted to the malaria epidemiological studies of the CISM/National Institute of Health 1996.

### Covariate data

The climate seasons are defined according to the rainfall and the temperature of the region during the period of study. Two seasons have been clearly distinguished: the wet season with high transmission of malaria from November to April and the dry season with low transmission of malaria between May and October. Three periods were created: the first from December 1996 to October 1997, the second from November 1997 to October 1998 and the third from November 1998 to July 1999. The sex and the age of the children were also divided into three groups: less than one year, from two to five years and above five years.

### Statistical analysis

The incidence rates of malaria were calculated as the number of new malaria episodes divided by the number of children week at risk. The incidence density of malaria was expressed as number of case per 1,000 children-weeks at risk (PWAR). The confidence intervals of the incidence density were calculated assuming that the number of new malaria episodes are Poisson distributed. A preliminary Poisson regression analysis was carried out to assess the relationship between the incidence of malaria and the covariates: sex, age groups, period, and climate season. The association between the covariables and the incidence was evaluated using the log-likelihood ratio test. This bivariate Poisson regression was fitted in the SAS programme with the GENMOD procedure [11]. The dependent variable is the count of new malaria cases detected per week in each neighbourhood, period, climate season, sex and age group and the offset of the Poisson regression is defined as the sum of week at risk per child. The incidence of malaria was then fitted according to the associated covariates in the preliminary regressions. The exponential of the coefficients of the covariates are equal to the incidence density ratio. This model is referred to as the non-spatial model. Thereafter, an exploratory data analysis was made to check the hypothesis of the existence of spatial association. The Moran's I test was used as an exploratory measure of spatial association [12]. The p-value associated with the Moran's I test was calculated by a Monte Carlo sample of 1,000 permutations.

The inclusion of regional random effects with a conditional autoregressive structure in the model for fitting the data would be called the spatial model [5]. The regional random effects were used at a neighbourhood level to take into account the spatial correlation present in the data. The spatial correlation was defined among neighbourhoods that had a common border. This regional random effect is a neighbourhood-specific random effect and assumes dependence on incidence density of the neighbourhoods and, concretely, induces 'local' smoothing by complete borrowing of strength from the neighbours. The exponential of the regional random effects is the neighbourhood-specific adjusted relative risk.

Next, this model was extended to include other regional random effects without spatial structure. This assumes independence on incidence density of the neighbourhoods and induces 'global' smoothing. With this spatial + non structured model, it is possible to check if part of the variability is not spatial [6]. Finally, seasonal random effects are considered. In this model the spatial correlation is nested within climate season, being a spatial seasonal model. Basically, the spatial effects nested within the climate season allow the spatial patterns at each season to be completely different [7].

The details of the models applied are explained in Additional file 1.

The parameters of the regression were estimated by the Bayesian methods using Gibbs Sampling (WinBUGS software, version 1.4 [13]) and three chains were run from dispersed starting values. Non informative prior distributions for the parameters were used. The convergence of the chains was assessed by the Gelman and Rubin statistic [14]. After convergence, a final sample of 5,000 interactions was collected to obtain summaries of the posterior distributions of the parameters. The models were compared using the deviance information criterion (DIC) suggested by Spiegelhalter *et al *[15]. The credibility intervals for the parameters estimated were calculated directly from the posterior distributions with a probability of 95%.

## Results

A total of 2,006 children were followed from December 1996 to July 1999. The average incidence density was six cases per 1,000 PWAR. The average seasonal rainfall and monthly mean temperature were of 1328.67 mm (SD: 374.59 mm per season) and 25.33°C (SD: 0.68°C) during the wet season and 372.10 mm (SD: 85.27 mm per season) and 20.80°C (SD: 0.58°C), respectively, in the dry season.

The crude incidence density for each level of sex, age, period and climate season are presented in Table 1. The likelihood ratio test for the covariate sex shows that the incidence of malaria is not different between males and females. In contrast, there is an association between the incidence and the group of age, period and climate season.

**Table 1:** Bivariate analysis of the relationship between malaria and covariates (sex, age, period, and climate season).

Covariates	Cases	Incidence density^(1)^	Confidence Interval 95%		Log-Likelihood ratio test	p-value
Sex						
Male	323	6.1	5.5	6.8	0.25	0.6188
Female	294	5.9	5.3	6.6		

Age						
Less than 1 year	259	6.7	5.9	7.5	25.16	< 0.001
From 2 to 5 years	287	6.7	6.0	7.5		
Greater than 5 years	80	3.8	3.0	4.7		

Period						
First (Dec 96 – Oct 97)	121	4.8	4.0	5.8	73.74	< 0.001
Second (Nov 97 – Oct 98)	173	4.2	3.6	4.9		
Third (Nov 98 – July 99)	324	8.9	7.9	9.9		

Climate Season						
Dry (May-Oct)	199	3.9	3.4	4.5	73.71	< 0.001
Wet (Nov-Apr)	419	8.1	7.4	9.0		

^(1) ^per 1000 children at risk per week

**Figure 3:**
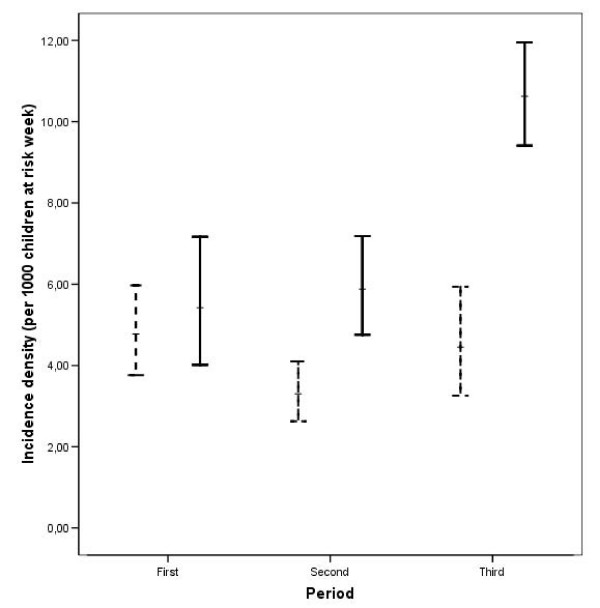
**Crude incidence density of malaria (and 95% confidence interval) according to climate season and period.** The incidence density of malaria is expressed as 1,000 children week at risk. Dashed line is the dry climate season and solid line is the wet climate season.

Figure 3 shows the confidence intervals of the incidence density of malaria according to the climate season and period. It can be observed that in the second and third periods the incidence density of the wet season may be greater than that of the dry season, and the magnitude of the differences between climate seasons may not be the same. This potential effect was introduced in the Poisson regression including an interaction effect of the period and climate season variables.

The incidence density for each neighbourhood is plotted in Figure 1. During the follow-up there were no cases in 19 neighbourhoods (16.5% of neighbourhoods) and the maximum incidence was 53.1 cases per 1,000 PWAR.

According to the exploratory data analysis with the Moran's I test (value = 0.214 p-value = 0.01), the hypothesis of the existence of spatial association is compatible with our data.

**Figure 4:**
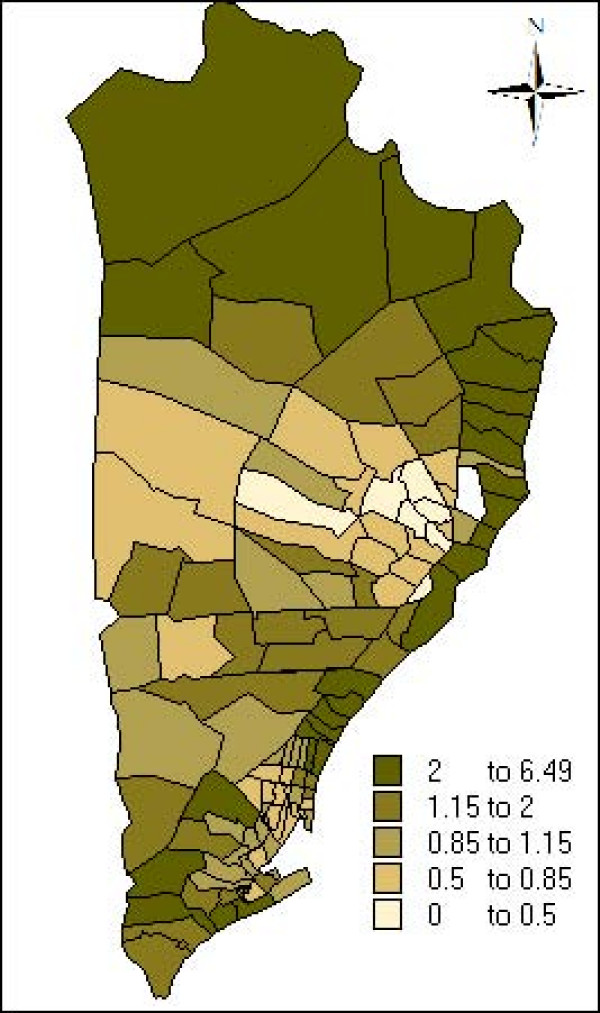
Neighbourhood-specific adjusted relative risk during dry climate season estimated by the spatio-seasonal model.

**Figure 5:**
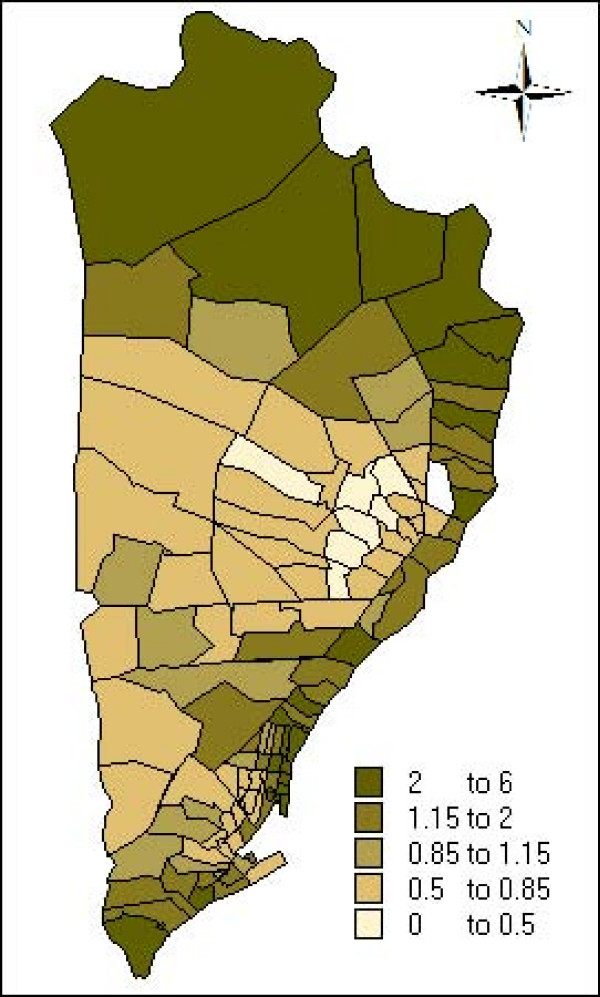
Neighbourhood-specific adjusted relative risk during wet climate season estimated by the spatio-seasonal model.

The estimate and the credibility intervals for the parameters of the non spatial model, spatial model, spatial+non structured model and spatial seasonal model are presented in table 2. In all the models the incidence of malaria is associated with age, period interval and the interaction between the climate seasons and the period. Figures 4 and 5 plot the neighbourhood-specific adjusted relative risk obtained in the spatial seasonal model for the dry and wet climate seasons, respectively. Although in the dry climate season some neighbourhoods in the centre of the region have a greater incidence of malaria, in both seasons, a similar spatial pattern is observed. The posterior probability that the regional random effects were different between the dry and wet seasons was also calculated, and it was found that only two neighbourhoods presented significant differences (data not shown). Apart from the random effects, the spatial variance of the dry season (spatial variance = 1.674; CI 95%: [1.178, 2.279]) did not present statistical differences with the wet season (spatial variance 1.222; CI 95%: [0.929, 1.570]). Indicating that the spatial variability of malaria incidence does not vary according to the climate season.

**Table 2:** Posterior estimates of regression coefficients and the variances related to random effects obtained by the different models

Coefficients	Non-Spatial Model	Spatial Model	Spatial Model + Non-structured random effects	Spatial-seasonal model
Age				
< = 1 year old	0.49 (0.22,0.73)	0.49 (0.22, 0.76)	0.49 (0.24, 0.77)	0.49 (0.24, 0.75)
(1 and 5 years)	0.59 (0.35, 0.75)	0.59 (0.35, 0.85)	0.56 (0.35, 0.86)	0.60 (0.34, 0.86)
Over 5 years	baseline	baseline	baseline	baseline

Season				
Wet	0.10 (-0.26, 0.45)	0.07 (-0.30, 0.42)	0.06 (-0.31, 0.42)	0.29 (-0.13, 0.73)
Dry	baseline	baseline	baseline	baseline

Period				
First	-0.39 (-0.71, -0.07)	-0.37 (-0.69, -0.05)	-0.37 (-0.70, -0.04)	-0.35 (-0.66, -0.02)
Second	-0.03 (-0.40, 0.33)	-0.04 (-0.42, 0.33)	-0.04 (-0.41, 0.32)	-0.03 (-0.42, 0.35)
Third	baseline	baseline	baseline	baseline

Season × Period				
Wet & Second	0.49 (0.02, 0.98)	0.49 (0.03,0.96)	0.49 (0.03, 0.98)	0.462 (-0.02, 0.94)
Wet & Second	0.76 (0.30, 1.25)	0.82 (0.33, 1.32)	0.82 (0.35, 1.30)	0.792 (0.32, 1.28)

Random effects				

Spatial variation	-	1.29 (0.68, 0.93)	1.26 (0.97, 1.59)	1.67 (1.18, 2.28)^1^
				
				1.22 (0.93, 1.57)^2^

Non-structured variation	-	-	0.18 (0.06,0.39)	-

DIC^3^	2333.65	2060.16	2061.59	2070.090

^1^spatial variance for the dry season, ^2 ^spatial variance for the wet season, ^3 ^DIC: Deviance Information Criterion. The smaller DIC value indicates a better fitting model. Credibility intervals at 95% in brackets

Model comparison showed that the spatial model had a small DIC value and is, therefore, the model that best fit the data. The spatial seasonal model, that incorporates regional random effects nested within climate seasons, had only a slightly higher DIC value. Similarly, when the spatial model is extended with non-structured random effects, the DIC value is also slightly higher. The spatial variance of this extended model (1.26, CI 95%: [0.97, 1.59]) is very similar to the variance of the spatial model (1.29, CI 95%: [0.68, 1.93]) and the non-structured variation is very close to 0 (0.18, CI 95%: [0.06; 0.39]. Thus, the extended model is not a better model than the spatial model and the main source of variability of these data is their spatial correlation.

According to the results under the spatial model, the malaria incidence in children of less than one year of age and those between one and five years of age is higher than that of children above five years of age (incidence density ratio = 1.648; CI 95%: [1.275; 2.113] and incidence density ratio = 1.828; CI 95% [1.415; 2.352], respectively).

During the first period the incidence of malaria in the wet season is not different to that of the dry season (incidence density ratio = 1.099 CI 95% [0.752; 1.566]). However, the incidences in the following two period intervals are greater in the wet than in the dry season. In the second period interval the density ratio is 1.775 (CI 95%: 1.298; 2.380) being slightly higher in the third period: incidence density ratio = 2.481 (CI 95%: 1.784; 3.346).

**Figure 6:**
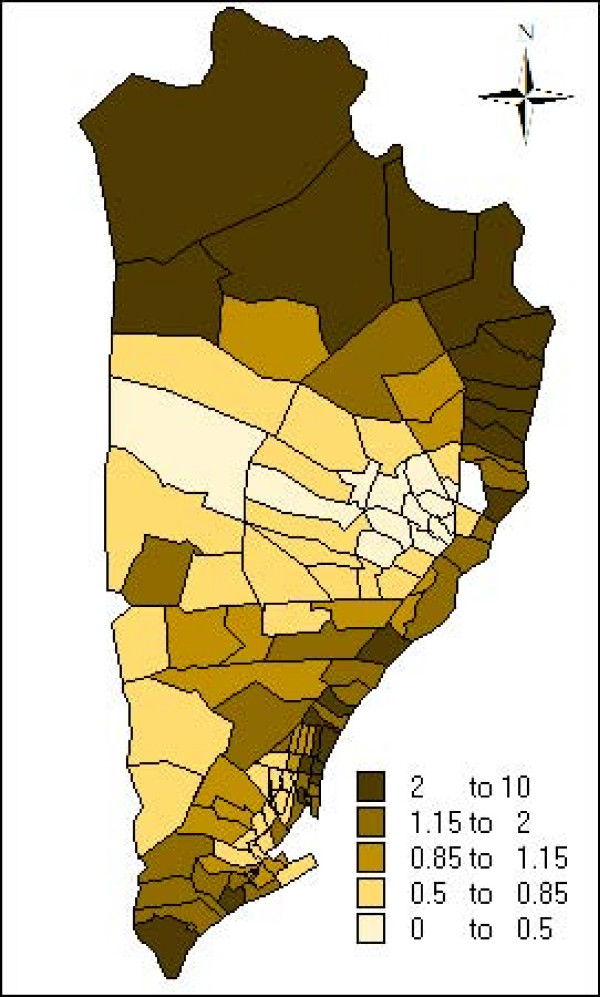
Neighbourhood-specific adjusted relative risk estimated by the spatial model for the neighbourboods of the district of Manhiça.

**Figure 7:**
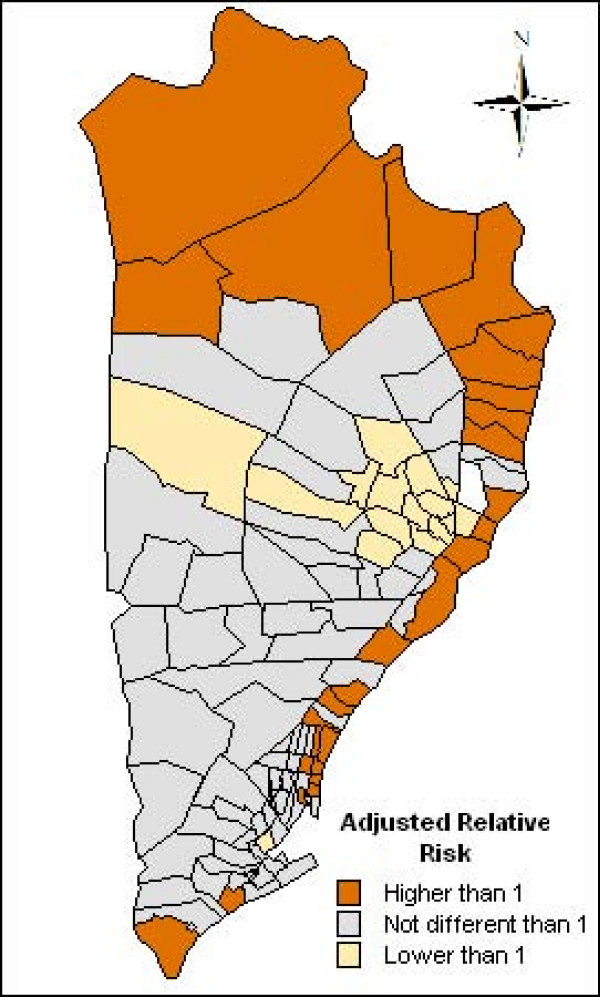
**Posterior probability of the neighbourhood-specific adjusted relative risk to be different than one estimated by the spatial model.** Orange colour: adjusted relative risk higher than one, grey colour: adjusted relative risk not different than one and yellow: adjusted relative risk lower than one.

To illustrate the spatial distribution of the incidence of malaria, Figure 6 maps the neighbourhood-specific adjusted relative risks, and Figure 7 maps the posterior probability that the neighbourhood-specific adjusted relative risk was different to one. This posterior probability was monitored with WinBUGS as the proportion of interactions of the final sample of 5,000 interactions, with a neighbourhood-specific adjusted relative risk was superior or inferior to 1. Thus, the regions with a posterior probability higher than 0.95 means that the adjusted relative risk was superior to one, while the regions with a posterior probability lower than 0.05, indicates that the adjusted relative risk was lower than one, and in other cases the adjusted risk relative is not different to one. It can be seen that the neighbourhoods with a higher incidence of malaria were in the North and Northeast, and the neighbourhoods with a lower incidence were in the center of the study area.

## Discussion

The objective of this study was to determine the variation that seasonal effects can produce on the spatial distribution of malaria in children under the age of 10 years living in an area of the Manhiça district. The results of this study confirm that the incidence of malaria in Manhiça has a clear spatial pattern, with a high incidence in the neighbourhoods situated in the north and northeast. The variation in climate season influences the incidence in malaria but does not modify the spatial patterns.

In this study, the incidence of malaria was obtained by a weekly follow up of a cohort of children in each neighbourhood. This method reinforces the reliability of the estimation of the incidence rates for each neighbourhood and eliminates the uncertainty involved in trials studying cases detected by passive detection methods.

There was no difference in the incidence of malaria between the dry and wet seasons in the first period (Dec 96 – Oct 97). In contrast, in the second (Nov 97 – Oct 98) and third (Nov 98 – July 99) periods there was a greater incidence in the wet season. The results of this first period may have been the consequence of this serial data since the standard effect is for the incidence to be higher in the wet season. The interaction between period and climate season shows that the climate season varies along years. In addition to the climate season and period, our model incorporated the demographic characteristics of the children. Hence, this model was able to obtain the associations between the incidence in malaria and the seasons, adjusting for confounding demographic effects. In relation to demographic factors, young children up to five years of age are at the highest risk of malaria infection, probably they have not yet developed sufficient immunity, making these early years particularly susceptible.

The covariates age group, period, and climate seasons, do not explain all the variability in the data, because there is a part of variability (overdispersion) that is captured by the regional random effects. It should also be taken into account that this overdispersion can be caused by the occurrence of more zero observations than expected by a Poisson distribution. To discard an excess of zeros in the data a zero inflated Poisson regression is also used [16]. However, this model has a higher DIC (2301.27) than the final model selected (DIC = 2060.160 l).

In contrast to the spatial-temporal studies performed in Zimbabwe [17], the spatial-seasonal model fitted used in this study allowed the analysis of the geographical distribution of malaria incidence during the climate seasons and comparison of the effects. In the study of the incidence in Zimbabwe the spatial distribution of the incidence was not associated with the time. Moreover, these authors studied whether the trend of the incidence changes in all the individual regions in the country.

According to these results, the neighbourhoods in the north and north-east, which had a higher risk of malaria, were located close to the Incomati River, concretely, on the edge of the swampy area of the river which is normally flooded during the wet season and seldom dries up after previous rainy season. In the study by Aranda *et al *[3], this area of high incidence coincided with the most abundant sites of mosquito breeding and with the greatest number of anopheline mosquitoes collected in the houses located therein. Among anophelines, anopholes funestus Giles represented 72.3%, being the most abundant species during all seasons. Saute *et al *[18] found that *P. falciparum *produced 90% of the cases of malaria in the region and the prevalence of infection by *P. falciparum *was higher in the north and northeast. The capacity of flight dispersion of the *An. funestus *(about 1 km – 2 km) [19] agreed with or justified that neighbourhoods that limited the swampy area presented higher incidences. Therefore, all these findings suggest that the risk of malaria could be related to proximity to the ecosystem vector of the *P. falciparum*.

## Conclusion

The incidence of malaria in Manhiça area presents a spatial pattern which is independent of the seasonal climatic conditions. The climate modifies the incidence of malaria in the entire region but does not change the spatial pattern of the incidence of this disease. Children under five years of age are at the highest risk of malaria infection.

These findings may be useful for the planning of malaria control activities. These activities can be performed taking account that the neighbourhoods with more incidence of malaria do not change over the annual climate seasons. Thus, these results suggest the need to plan interventions which continue over time in the areas with the highest incidence to control the transmission and thereby maximize the reduction in incidence.

## Competing interests

The authors declare that they have no competing interests.

## Authors' contributions

RA and CA were responsible for statistical analysis and the preparation of the manuscript. JA, FS and PA conceived and designed the study. FS, DN and AN were responsible for fieldwork and the geographical information system of Manhiça. JA was responsible for data cleaning and manuscript preparation.

## Supplementary Material

Additional file 1 Description of the model formulation in terms of the malaria data set. Click here for file
