# Supplementary material for: Spatio-seasonal modeling of the incidence rate of malaria in Mozambique
Source: Malar J. 2008 Oct 31;7:228. doi: 10.1186/1475-2875-7-228 (PMC2584655; doi:10.1186/1475-2875-7-228)
Supplement: Additional file 1 — Description of the model formulation in terms of the malaria data set. [file 1475-2875-7-228-S1.pdf]

Additional file 1:

We describe our model formulation in terms of the malaria data set. Thus, let n_{itsk} denote the sum of weeks at risk of the children in each neighbourhood *i* (i=1,…115) during the period *t* (t=1,2,3) and climate season *s* (s=1,2) for a specific age group *k* (k=1,2). We assume that the number of new malaria cases per week Y_{itsk} in each neighbourhood *i* during the period *t* and climate season *s* and age group *k* has a Poisson distribution with mean $\mathbf{n_{itsk}\exp(\mu_{itsk})}$.

The non-spatial model is defined as:

$$\log(\mu_{itsk}) = \log(n_{itsk}) + \beta_0 + \beta_t + \beta_s + \beta_{ts} + \beta_k$$

where β_t is the effect of period *t*, β_s, is the climate season effect, β_{ts} is the period *t* effect at climate season *s*, and β_k is the age group effect.

In the spatial model, the regional random effects b_i is incorporated into the linear predicted, thus, $\log(\mu_{itsk})$ is defined as:

$$\log(\mu_{itsk}) = \log(n_{itsk}) + \beta_0 + \beta_t + \beta_s + \beta_{ts} + \beta_k + b_i$$

For this spatial structured component, we chose a simple Gaussian intrinsic auto regression. Thus, the conditional distribution of b_i is

$$b_i \mid b_{-i} \sim N\left(\bar{b}_i, \sigma_b^2 / m_i\right)$$

where $\bar{b}_i$ is the corresponding mean value over the m_i neighbourhoods that are geographically contiguous to *i* and σ_b^2 is a spatial variance parameter.

The exponential of the regional random effects (e^{bi}) is the neighbourhood-specific adjusted relative risk.

The spatial+ non structured model, is that which includes the regional random effects prior to b_i, and other regional random effects without spatial structure, θ_i. It is assumed that $\theta_i \sim N(0, \sigma_\theta^2)$ where σ_θ^2 is the non-structured variance. Thus, $\log(\mu_{itsk})$ is defined as:

$$\log(\mu_{itsk}) = \log(n_{itsk}) + \beta_0 + \beta_t + \beta_s + \beta_{ts} + \beta_k + b_i + \theta_i$$

Finally, in the spatial-seasonal model, the regional random effect is nested within climate season. This effect is written as $b_i^{(s)}$. That is, $b_i^{(s)}$ is a random effect for the i*th* region in climate season *s*.

For this spatial structured component, we also chose a simple Gaussian intrinsic auto regression. Thus, the conditional distribution of $b_i^{(s)}$ is

$$b_i^{(s)} \mid b_{-i}^{(s)} \sim N\left(\bar{b}_i^{(s)}, \sigma^2_{b^{(s)}} / m_i\right)$$

where $\bar{b}_i^{(s)}$ is the corresponding mean value for the climate season *s* over the m_i neighbourhoods that are geographically contiguous to *i* and $\sigma^2_{b^{(s)}}$ is a spatial variance parameter for the climate season *s*. In this situation $\log(\mu_{itsk})$ is defined as:

$$\log(\mu_{itsk}) = \log(n_{itsk}) + \beta_0 + \beta_t + \beta_s + \beta_{ts} + \beta_k + b_i^{(s)}$$
